# Phase-Change Materials in Concrete: Opportunities and Challenges for Sustainable Construction and Building Materials

**DOI:** 10.3390/ma15010335

**Published:** 2022-01-03

**Authors:** Raju Sharma, Jeong-Gook Jang, Jong-Wan Hu

**Affiliations:** 1 Division of Architecture and Urban Design, Urban Sciences Institute, Incheon National University, 119 Academy-ro, Yeonsu-gu, Incheon 22012, Korea; rsharma@thapar.edu; 2 Department of Civil and Environmental Engineering, Incheon National University, 119 Academy-ro, Yeonsu-gu, Incheon 22012, Korea; jongp24@inu.ac.kr; 3 Incheon Disaster Prevention Research Center, Incheon National University, 119 Academy-ro, Yeonsu-gu, Incheon 22012, Korea

**Keywords:** phase change materials, concrete, building envelopes, carbon emission, United Nations sustainable development goals, thermal energy storage

## Abstract

The use of phase-change materials (PCM) in concrete has revealed promising results in terms of clean energy storage. However, the negative impact of the interaction between PCM and concrete on the mechanical and durability properties limits field applications, leading to a shift of the research to incorporate PCM into concrete using different techniques to overcome these issues. The storage of clean energy via PCM significantly supports the UN SDG 7 target of affordable and clean energy. Therefore, the present study focuses on three aspects: PCM type, the effect of PCM on concrete properties, and connecting the outcome of PCM concrete composite to the United Nations sustainable development goals (UN SDGs). The compensation of reduction in strength of PCM-contained concrete is possible up to some extent with the use of nanomaterials and supplementary cementitious materials. As PCM-incorporated concrete is categorized a type of building material, the large-scale use of this material will affect the different stages associated with building lifetimes. Therefore, in the present study, the possible amendments of the different associated stages of building lifetimes after the use of PCM-incorporated concrete are discussed and mapped in consideration of the UN SDGs 7, 11, and 12. The current challenges in the widespread use of PCM are lower thermal conductivity, the trade-off between concrete strength and PCM, and absence of the link between the outcome of PCM-concrete composite and UN SDGs. The global prospects of PCM-incorporated concrete as part of the effort to attain the UN SDGs as studied here will motivate architects, designers, practicing engineers, and researchers to accelerate their efforts to promote the consideration of PCM-containing concrete ultimately to attain net zero carbon emissions from building infrastructure for a sustainable future.

## 1. Introduction

The building sector is one of the significant contributors across the globe to the resiliency and safe livelihoods of humans [1]. Nevertheless, the building sector consumes numerous natural resources and conventional power in every part of the world and has, therefore, been characterized as contributing to the degradation of the environment. The six largest emitters of greenhouse gases (GHG), together accounting for 62% globally, are China (26%), the United States (13%), the European Union (more than 8%), India (7%), the Russian Federation (5%), and Japan (almost 3%). In 2018, the largest increase in GHG emissions was reported for India (+5.5%), followed by the United States (+2.7%) and China (+1.9%) [2]. An analysis of developing countries reveals greater energy demand in the future [3]. For instance, the international energy agency (IEA) has anticipated that by 2035, India’s energy demand will have increased to 8.6% of global demand, or 1464 Mtoe [4]. Additionally, energy consumption by commercial buildings is predicted to expand at a faster rate (8%) than that of the residential sector (5%), and floor space is predicted to grow from 659 million square meters in 2010 to 1900 million square meters in 2030 in India [5]. However, up to 2035, China is projected to retain its share of energy consumption at around 23% out of global primary energy consumption, though after 2035 energy demand in China will gradually decline [2].

**Figure 1:**
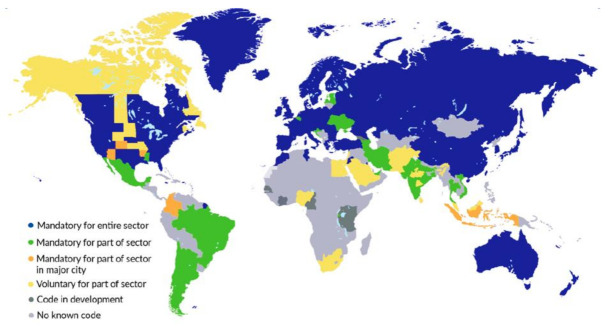
Building energy code by jurisdiction, 2018–19 (IEA, All right reserved) [8]. Note—this map is without prejudice the status of or sovereignty over any territory, to the delimitation of international frontiers and boundaries and to the name of any territory, city or area.

In context to global building requirements, over the next decade, more than 20% of expected global building additions up to 2050 will be built, and more than 50% of the corresponding floor area additions will occur in regions that currently do not have mandatory energy codes in place for the entire building sector [5]. The upcoming infrastructure projects can follow the design and guidelines to make a building completely energy efficient [6]. However, for the built environment (noncompliance with existing energy codes), the fields of rehabilitation and retrofitting become important. Figure 1 shows the present status of building energy codes in different countries. In the future, to evade the expensive green retrofits of buildings that are noncompliant with existing energy codes, the immediate effective implementation of building energy codes is required [7,8]. This is important because an absence of energy codes means users and contractors when building the infrastructure may not do so on the basis of the vision incorporated in the UN SDGs.

**Figure 2:**
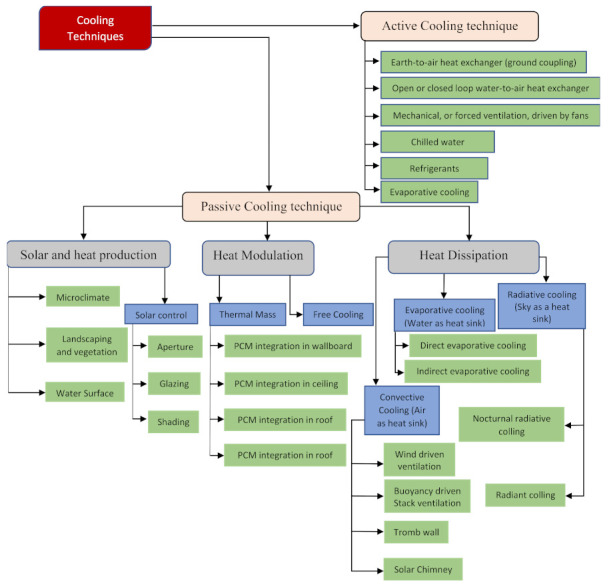
Various active and passive cooling techniques [16,17].

Seventeen SDGs were adopted by the UN in 2015 [9]. Particularly, UN SDGs 7 (affordable and clean energy), 9 (industry, innovation and infrastructure), 11 (sustainable cities and communities), and 12 (responsible consumption and production) are closely related to infrastructure development. Therefore, SDG intervention is a mandatory achievement for resilience and sustainability through infrastructure building [10]. The transition from fossil fuels to renewable energy to attain sustainability considering that most existing practices are based on fossil fuels is a major challenge for practitioners in the field of infrastructure development [11]. Similarly, thermal comfort (the energy demand for cooling, heating and air conditioning) in a building envelope is significantly based on the utilization of fossil fuels, and this existing practice violates the UN SDGs [12]. In the building sector, 60% of the total energy is consumed by heating, ventilation, and air conditioning (HVAC) systems for climate control [13,14]. Broadly, active, passive and hybrid cooling strategies facilitate indoor thermal comfort. Indoor thermal comfort significantly affects the health and productivity of the occupants of a building [15]. Overall, the passive cooling technique is considered to be a sustainable technique to meet current thermal demands due to its use of renewable sources of energy [16]. Therefore, relying only on cooling systems powered by conventional energy has resulted in an increase in the demand for fossil fuel-based energy in the future, hindering the global establishment of thermal energy storage based on renewable energy. Various recently developed impeccable renewable energy passive cooling technologies to make building envelopes sustainable have been innovated and broadly classified into heat protection, heat modulation and heat dissipation techniques [17]. The detailed classification of active and passive cooling techniques is presented in Figure 2.

Over the last decade, thermal energy storage (TES) has been a promising technology to achieve a low-carbon future [18]. In fact, the application of phase-change materials (PCM) to enhance the TES for thermal comfort is widely accepted [19]. PCM is recognized as a potential game changer in the field of sustainable infrastructure development and is also feasible for built environment sustainability [20]. Therefore, the use of PCM in the building can be a tradeoff between the future energy demand and the goal of reducing fossil fuel consumption to promote infrastructure related to the UN SDGs.

The successful application of PCM in building to attain thermal comfort has been reported [21,22,23,24]. A simulation of PCM building bricks reported by the Alawadhi [25] revealed that the PCM placement at the centerline location of PCM cylinders is best in terms of thermal effectiveness while also helping to maintain the strength of the bricks. A study conducted by Zhuang et al. [26] reported that PCM placed close to the inner side of a hollow block outperformed in terms of thermal performance. However, the use of PCM with different placements in concrete blocks enhances the thermal performance to a different extent depending on its use. In addition, the interaction between PCM and cementitious materials is an issue of concern. Accordingly, different techniques of PCM inclusion in building materials, such as alveolar bricks, hollow bricks and CSM panels, have been tested [27]. Therefore, different non-contact incorporation methods of PCM with concrete are attracting much attention among sustainable building researchers [28,29]. Eventually, PCM incorporation in construction materials will support the UN SDGs, but no attempts have been made to correlate the contributions of PCM concrete composites to realize the UN SDGs.

Therefore, the present study is an attempt to connect the contributions of PCM composite concrete as a building material with the UN SDGs. The use of PCM undoubtedly affects the architectural planning, material selection, execution, repair and rehabilitation, and demolition waste-management stages associated with building lifetimes. Efforts are made to address the impact of the use of PCM on the different stages of building lifetimes in this study as well.

## 2. Phase Change Materials

**Figure 3:**
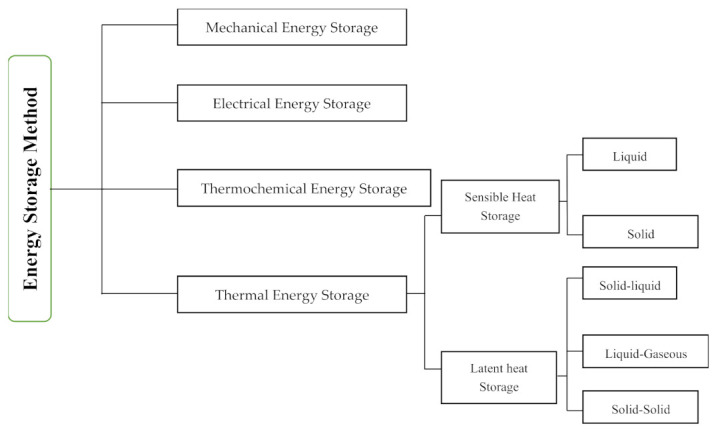
Different type of energy storage method [31,32].

**Figure 4:**
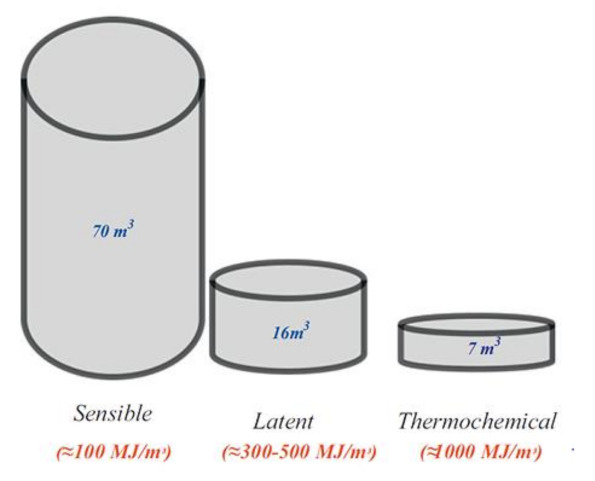
Volume needed to full cover the annual storage needs of an energy efficient passive house (6480 MJ) [33].

Before defining PCM holistically, a brief explanation of alternative energy storage methods would be beneficial. Ultimately, energy storage is a top priority for reducing fossil fuel consumption, but a sustainable method of energy storage must be practical and long-lasting. Figure 3 shows the different method for the storage of energy. Energy storage methods are categorized into four different types: mechanical energy storage, electrical energy storage, thermochemical energy storage, and thermal energy storage. The thermal energy storage type is a practical method in line with the United Nations SDGs following the consumption of clean energy for thermal comfort in the building sector. Thermal energy can be stored via a change in the internal energy of a material as sensible heat, latent heat, and thermochemical heat, or a combination of these [30,31,32]. The thermochemical energy storage method demonstrates the highest heat storage density compared to the latent heat and sensible heat storage methods, as shown in Figure 4. However, poor performance in terms of the heat and mass transfer of the thermochemical energy storage method is reported when experimental tests are run [33].

**Figure 5:**
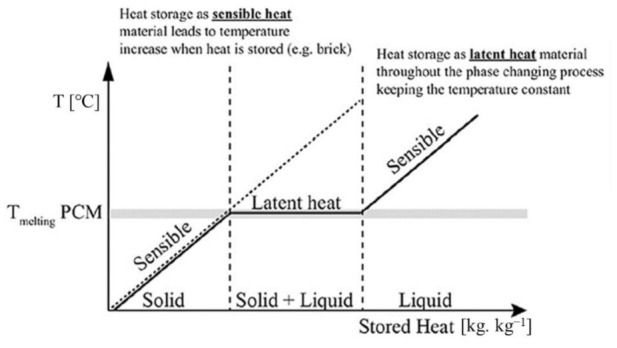
Latent heat storage for the case solid–liquid [41].

Furthermore, a comparison between latent heat storage (LHS) and sensible heat storage (SHS) shows that the thermal energy storage density of latent heat storage is higher than that of sensible heat storage [34,35]. Additionally, the temperature range of LHS required to store and release heat is narrower than that of SHS [36,37,38]. A large amount of latent heat is absorbed/released by the PCM during the process of transforming the physical properties, i.e., from a solid to a liquid or a liquid to a solid [39,40,41]. Unlike conventional (sensible) storage materials, PCM absorbs and releases heat at a nearly constant temperature, as shown in Figure 5.

**Figure 6:**
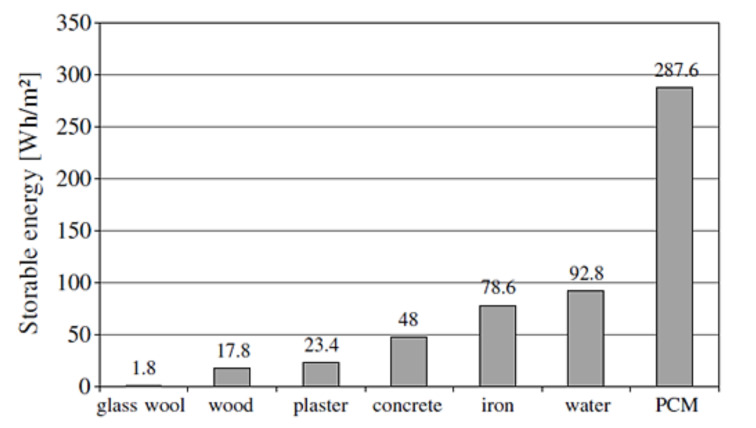
Maximum storable energy between 18 °C and 26 °C for 10 mm of material and for 24 h [42].

PCM can store five to fourteen times more heat per unit volume than sensible storage materials (water, masonry, or rock) [42,43,44]. Kuznik et al. [42] conducted a simulation using their in-house software CODYMUR to optimize the wallboard. The outcome of the experimental study sought to achieve the optimum thickness of PCM. A thickness of 10 mm of PCM was reported to be an optimal thickness. Furthermore, energy storage capabilities of 10 mm-thick PCM were compared with those of other materials, as shown in Figure 6. It was observed that latent-heat-based PCM shows enhanced storable energy capabilities, improving the thermal energy storage capacity. Therefore, LHS-based PCM is reported as the most practically relevant thermal storage material for building applications.

**Figure 7:**
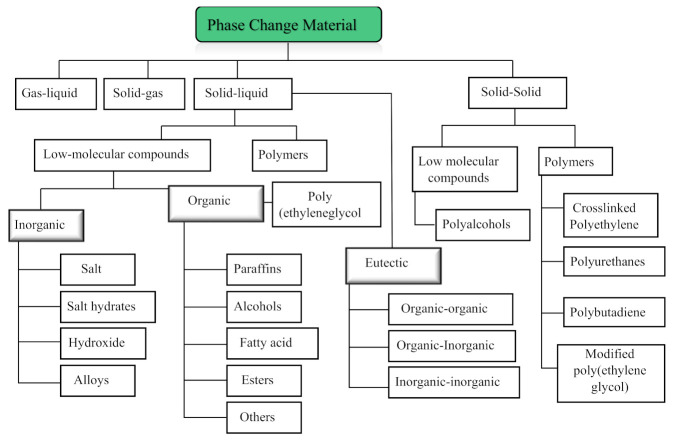
Classification of phase change materials [45,46,47].

Figure 7 shows the various types of PCMs. Among these PCMs, organic, inorganic and eutectic types are used broadly in buildings [45,46]. Specifically, the selection of a PCM depends upon its temperature range. A temperature range of 20–32 °C is the accepted range for passive heating and cooling of a building with PCMs [21,47]. A detailed discussion of PCMs is provided in the subsections below.

### 2.1. Organic PCMs

Organic PCMs can be either paraffin or non-paraffin type [48]. Paraffin is usually a mixture of straight-chain n-alkanes with the general formula CH_3_-(CH_2_)_n_-CH_3_ [47]. Paraffins are excellent energy storage and passive cooling PCMs due to several inherent properties: high latent fusion heating, a wide temperature range of transitional availability, a low cost, and desirable physical and chemical properties, such as congruent fusion with little or no therapeutic hysteresis [31,49]. Organic PCMs are more chemically stable and melt congruently, meaning that super-cooling is not a significant problem [50]. However, organic PCM exhibits low thermal conductivity, making the rate of heat storage/release of organic PCM low [51,52,53,54]. Acid (CH_3_(CH_2_)_2n_COOH) is the primary organic non-paraffin PCM [55]. Its melting point is comparable to that of the paraffin PCM, and it has excellent melting and freezing characteristics. However, the cost of non-paraffin PCMs is nearly three times that of paraffin PCMs [50]. Fatty acids and their acid esters and alcohols, glycols, etc., are non-paraffin types used as PCMs, and among these, fatty acids are the most commonly used PCMs in building applications for thermal storage [56]. The latent heat and melting properties of the various PCMs used in buildings are shown in Table 1.

### 2.2. Inorganic PCMs

Inorganic PCMs are salt hydrates (MnH_2_O), nitrates, and metallic materials having a slightly high heat of fusion. Some of their properties, such as high volumetric latent heat storage capacities, non-flammability, and high thermal conductivity make these materials appropriate for building applications. Furthermore, they are inexpensive and readily available [57]. However, some of the disadvantages are corrosion to most metals, phase separation and segregation, a lack of thermal stability, and supercooling during the solid–liquid transition process [50,58]. Super-cooling is defined as a “delay in the start of solidification,” taking place whenever a PCM undergoes a phase change from a liquid to a solid [59]. As a result, for full utilization of the latent heat, a large temperature difference between charging and discharging is required, which is undesirable for efficient energy storage applications [60]. Specifically, a realistic possibility for minimizing supercooling is to introduce sufficiently large PCM microcapsules, but the interaction with the large diameter of the PCM often breaks down during the mixing process when using these materials in building applications [61,62]. Another concern with inorganic PCMs is their degradation and breakdown after repeated phase-change cycles. Among all inorganic PCMs, salt hydrates are widely studied for thermal energy storage.

### 2.3. Eutectic PCMs

Eutectics are organic and/or inorganic compound mixtures [63,64,65]. As a result, eutectics can be created as organic–organic, inorganic–inorganic, or organic–inorganic mixtures [66]. Eutectic PCMs have a higher density than organics and a single melting temperature and therefore do not separate into components during a phase change [67]. As a result, phase separation and super-cooling are not observed in these materials. Eutectics usually have a high thermal cycle, particularly in comparison to salt hydrates (inorganic PCM). The eutectic PCM offers the flexibility of customizing the melting temperature and enthalpy according to the actual field application. Therefore, eutectic PCMs with lower melting peak temperatures have received substantial attention in the building sector [65]. Mert et al. [68] encapsulated a mixture of capric acid and oleic acid containing hexadecane in styrene–divinylbenzene copolymer shells via an emulsion polymerization technique. The obtained melting point range and enthalpy were 14.1–24 °C and 123 J/g, indicating suitability for thermal storage in building applications. Further, recent research is focused on thermal conductive enhancements of eutectic PCMs using thermal conductive fillers (carbon-based materials and nanoparticles) [69,70]. Some of these eutectic mixtures with their melting points and latent heat values are depicted in Table 1.

**Table 1:** Melting point and latent heat of different types of (a) organic [31,71,72,73,74], (b) inorganic [75,76,77], and (c) eutectic [64,65,78,79,80,81] type of PCM.

Organic PCM			Inorganic PCM			Eutectic PCM		
PCM Type	Melting Point (°C)	Latent Heat of Fusion (kJ/kg)	PCM Type	Melting Point (°C)	Latent Heat of Fusion (kJ/kg)	PCM Type	Melting Point (°C)	Latent Heat of Fusion (kJ/kg)
Paraffin C17	21.7	213	K_2_HPO_4_·6H_2_O	14	109	Capric Acid/Palmitic Acid/Stearic Acid	19.83	154.11
Paraffin C 18	28	244	LiNO_3_·2H_2_O	30	296	Hexadecanol/Palmitic Acid/Lauric Acid	26.527	179.63
Butyl stearate	19	140	LiNO_3_·3H_2_O	30	189	Lauric/Palmitic/Steric Acid	36.79	159
Emerest 2325	20	134	FeBr_3_·6H_2_O	27	105	Capric Acid/Lauric Acid/Palmitic Acid	20.75	134
Emerest 2326	20	139	CaCl_2_·12H_2_O	29.8	174	Capric Acid/Myristic Acid/Palmitic Acid	17	131.7
Tetradecane (C_14_H_20_)	5	172.21	CaBr_2_·6H_2_O	34	138	Oleic acid/Isopropyl Palmitate/Butyl stearate	5.14	104.12
Paraffin waxes RT45	45	160	LiClO_3_·3H_2_O	8	253	53% Mg(NO_3_)_2_·6H_2_O + 47% AL(NO_3_)_2_·9H_2_O	66	168
Paraffin waxes RT55	55	170	KF·4H_2_O	19	231	14% LiNO_3_·3H_2_O + 86% Mg(NO_3_)_2_·6H_2_O	72	180
Formic acid	7.8	247	Na_2_SO_4_·10H_2_O	32.4	248	55% CaCl_2_·6H_2_O + 45% CaBr_2_·6H_2_O	14.7	140
Glycerin	17.9	198.7	Na_2_CO_3_·10H_2_O	33	247	-	-	-

## 3. PCM Incorporation in Concrete

**Figure 8:**
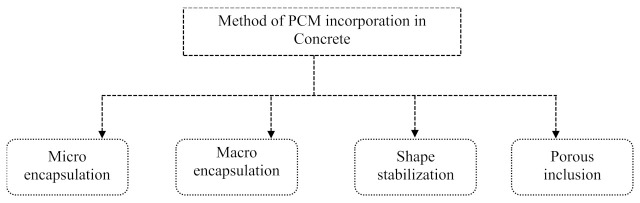
Four different methods of incorporating PCM in concrete.

Figure 8 shows the four different methods of PCM incorporation in concrete. These four methods of PCM incorporation in concrete and their performance capabilities in terms of their thermal, mechanical, and durability properties are outlined in this section. The selection of the PCM type and the associated method of incorporation in concrete significantly influence the overall performance of the concrete. Therefore, this section is prepared to assist designers and executive engineers to enrich their decisions while selecting PCMs for thermal storage applications in concrete.

Microencapsulation is a method of placing the PCM in a polymeric shell. The hollow capsules made of a polymeric shell filled with PCM were manufactured between 1 and 300 µm sizes [82]. The outer shell acts as a container for the PCM. Therefore, PCM can store as a core material in liquid or solid form in the polymer shell. The PCM having a phase change temperature between −10 °C to 80 °C is suitable for manufacturing the microcapsules [83]. The microencapsulated PCM (MPCM) was used successfully in the walls, roof, floors, gypsum wallboards, plaster, and mortar [84]. However, the demerits associated with MPCM are the low stiffness and strength compared to the ingredients of cement-based building materials. In addition, the leakage of MPCM in concrete during mixing degrades the mechanical properties of concrete [85]. The reduction in strength was due to the direct interaction of PCM with the cement matrix [86]. Macro encapsulation technique is similar to microencapsulation, but the size of the container for preserving the PCM is large such as a tube, spheres, or panels. The size of these containers is generally greater than 1 cm [81]. The component prepared using this technology can directly serve as a building element. However, some of the issues like leakage, poor heat transfer characteristics, and solidification at edges are the demerits of the macro encapsulation technique [87]. The third technique is shape stabilization focused on PCM preparation using supporting material. High-density polyethylene and styrene–butadiene–styrene are the common supporting material for manufacturing the shape-stabilized PCM. The mixture of PCM and supporting material melted and then mixed properly at high temperature. The PCM and supporting material mixture allow to cool down below the glass transition temperature of the supporting materials until it becomes solid. The advantage of this PCM is large apparent specific heat for phase change temperature region, stabilized shape during the phase change process, and no requirement of container [88]. This processing prevents the PCM leakage, but the lower thermal conductivity of the shape stabilized PCM limits its wide adaptability [89]. The porous inclusion technique follows the impregnation of PCM into the porous inclusions, and that composite material can be used in concrete for improving the thermal insulation [90]. Among these techniques, the research on PCM-filled lightweight aggregate and its effect on mechanical and thermal performance has been widely conducted [91,92]. Hitherto, concrete was specifically designed to obtain robust mechanical properties and durability. However, the concept of tailored concrete accelerated the production of a variety of concrete in the infrastructure industries. PCM-integrated concrete is also a type of tailored concrete, precisely developed to enhance the thermal properties (e.g., thermal conductivity, specific heat capacity, thermal diffusivity). However, the use of PCM in concrete via micro encapsulation, macro encapsulation, shape stabilization and porous inclusion technique decreases the strength and density of the concrete and increase the porosity [93]. Specifically, the leakage of PCMs in the liquid state [86,94,95,96,97], and interference of PCMs with cement hydration is the cause of strength reduction [98,99,100]. Therefore, the present study gathered information and presented an overview from the published literature with regard to PCM incorporation methods, procedures of incorporation into the matrix, the percentage of PCM use in the matrix, and effect of incorporation of PCM on the different properties of mortar/concrete, as summarized in Table 2. Accordingly, this field remains open for further investigations to realize safe, durable and PCM-compatible concrete.

Improvements in the thermal performance of concrete have been achieved using PCMs in concrete irrespective of the method adopted among the four described in Figure 8. The major impact of using PCM in concrete was observed on the mechanical performance of concrete, as presented in Table 2. The decrease in the overall mechanical properties of concrete is due to the chemical reaction and physical interaction with concrete materials [101,102,103]. The interactions of PCM and cement hydration products result in a decrease in the calcium silica hydrate crystallization in the cement matrix and produce a weaker phase in the cement binding system [36,86]. Therefore, the safe deposition of PCM into a lightweight aggregate along the coating of supplementary cementitious materials onto the outer surface can have the least negative effect on the concrete mechanical properties [29,104].

One fact that cannot be ignored is that the feasibility of PCM-incorporated concrete on a large scale in a field application is only possible when PCM has a neutral effect or minimal effect on the mechanical properties of concrete. The mass application of PCM-incorporated concrete can significantly decrease the fossil-fuel-based energy demand and contribute to the realization of the UN SDGs. Therefore, in the upcoming section, the possible benefits of using PCM in infrastructure, the mapping between PCM concrete composites and UN SDGs, and expected alterations in the traditional infrastructure practices are addressed.

**Table 2:** Different method of incorporating the PCM in concrete/mortar and their performance outcome.

Ref.	Material Type/Method of PCM Incorporation	PCM Name	Replacement/Addition	Properties Investigated	Performance
					(Improvement/Decrement)
[93]	Mortar/Micro encapsulation	Paraffin based microencapsulated PCM (Micronal DS 5040X)	Sand replaced with 1%, 3% and 5% PCM by mass	Isothermal calorimetry	Increment and delay in maximum rate of heat of hydration compared with the control mix.
				Thermogravimetry	Dehydration of C-S-H and dehydroxylation of CH were observed in the range of 35 °C to 540 °C.
				Differential scanning calorimetry	Higher weight loss was observed throughout the temperature range of 50 °C to 800 °C
				Thermal conductivity	Reduced with the increment of PCM in mortar.
				Compressive Strength	Soft microencapsulated PCM increases the porosity, so strength decreases.
[105]	High Performance hybrid fiber engineered cementitious composites/Microencapsulation	Micro PCM (Maximum particle size up to 300 μm)	Silica sand replaced with micro PCM by 1%, 2%, 3% and 5% by weight	Compressive strength	16.39% reduction at 28 days of ECC having 5% PCM.
				Thermal conductivity	Increase in the amount of micro PCM displays very little change between 0.90 and 1.1 W/mK
				Specific heat value	Increases the phase change enthalpy in the interval (56–72 °C) which, in turn, results in a thermal absorption capacity increase.
[106]	Ultra-high-performance concrete (UHPC)/Microencapsulation	Paraffin wax based micro encapsulated PCM (MPCM)	5% and 10% MPCM by weight of binder added in the mix	Compressive strength	Compressive strength of UHPC decreases because of the low strength of microcapsules and leakage in mortar while mixing.
				Thermal conductivity	Decreases with the increase of MPCM content.
				Heat storage capacity	Increase in the heat storage capacity determined by differential scanning calorimetry.
				Mercury intrusion porosimetry (MIP)	Capillary and transition pore volume increases sharply with the incorporation of MPCM, but the proportion of gel and large pores is relatively small.
[86]	Self-compacted concrete/Micro-encapsulation	Mixture of paraffin waxes in powder form, encapsulated in polymethyl methacrylate microcapsules, Micronal (DS 5008 X)	1%, 3% and 5% by mass of concrete added in the self-compacted concrete.	Compressive strength	Increasing PCM dosages lead to significantly lower the compressive strengths.
				Thermal conductivity	The addition of PCM particles into the mass of the concrete results in a reduction of thermal conductivity.
				Specific heat capacity	Increasing the amount of PCM in the mixture increases significantly its specific heat capacity (up to 3.5 times for the 5% PCM content).
[82]	Mortar/Microencapsulation	n-octadecane (C_18_H_38_) impregnated in the ceno-spheres	Mortar (reference mix) without CenoPCM Mortar without sealed CenoPCM. Mortar with sealed CenoPCM	Compressive strength	Mortar without CenoPCM (Reference mix) attained 50.80 MPa strength at 28 days of curing. Mortar without sealed CenoPCM attained 42.87 MPa strength at 28 days curing. Mortar with sealed (silica sol) CenoPCM exhibited 47.96 MPa strength at 28 days of curing. The lowest strength in the mortar containing unsealed CenoPCM could be partially attributed to the PCM absorbed on the shell surface.
[107]	Structural-functional integrated Concrete/Macroencapsulation	Commercial inorganic PCM (Rubitherm SP22) impregnated in lightweight aggregate (LWA)	PCM-lightweight aggregate (LWA) as partial replacement (25 and 50% by volume) of coarse aggregate	Compressive strength	Increasing PCM-LWA in a mix cause in a reduction in strength.
				Thermal performance of PCM-LWA panel	PCM-LWA has the ability to reduce the energy consumption by reducing the indoor temperature and shifting the loads away from the peak periods.
[108]	Structural-functional integrated Concrete/ Macro-encapsulation	Macro-encapsulated paraffin lightweight aggregate (LWA).	7%, 33% and 50% macro-encapsulated paraffin–LWA by volume of normal weight aggregate	Compressive strength	Compressive strength of macro encapsulated paraffin lightweight aggregate contained concrete is higher than the only LWA contained concrete.
				Thermal Performance	Rooms with macro-encapsulated paraffin–LWA show a lower indoor temperature as compared to the control room model during the heating and cooling process.
[109]	Concrete/Macroencapsulation	Paraffin octadecane (PO)	Replacing 25%, 50%, 75% and 100% of gravel normal coarse aggregate with PO filled hollow steel balls	Compressive strength	PCM contained concrete strength decreased with the increases in PCM content in the mix.
				Thermal performance of macro encapsulated PCM-HSB incorporated concrete panel	PCM contained concrete panel efficiently reducing the peak indoor air temperature in the range of 25–33% compared to control specimen.
[110]	Macro PCM contained hollow fire clay brick wall/Macro encapsulation	Metal steel macro-capsules filled with organic paraffin	-	Thermal performance	High thermal amplitude reduction of the wall specimens with PCM compared with the wall without PCM.
[90]	Light weight aggregate (LWA) used as PCM host.	paraffin-based liquid PCM	5% PCM by overall volume	Thermal conductivity	PCM impregnated LWA mortars show thermal conductivities 10% to 20% lower than the regular LWA (water-saturated) mortars, at a total PCM content of 5% by volume in the mortars.
				Compressive strength	The PCM contained LWA perform equivalent to water contained LWA due to the no leakage of PCM from the LWA during heat storage and released.
[111]	PCM impregnated light weight aggregate (LWA)/Porous inclusion	-	-	Differential scanning calorimetry	The EP/erythritol composite prepared by the vacuum impregnation treatment had the largest latent heat, which was 83% of that of pure erythritol.
				Cyclic stability test	The latent heat storage decreases as the cycles of heating and cooling increased.
					Without cycle—334.4 kJ/kg
					First Cycle—295.2 kJ/kg
					Second Cycle—238.1 kJ/kg
[112]	cement mortar/Porous inclusion	Paraffin	Sand replaced by PCM at the percentage of 20, 40, 60 and 80 by volume.	Compressive strength	28 days strength at replacement levels of 20%, 40%, 60% and 80% are 12%, 33%, 53%, and 70% lower than the control specimen, respectively.
				Apparent density	Decreases with the increasing replacement level of composite PCM.
				Thermal conductivity	Decreased with the increasing replacement levels of composite PCM.
				Thermal energy storage capacity	80% contained PCM mortar mix exhibited a maximum energy storage capacity of 125 kJ/kg, compared to 47 kJ/kg in NC.
[113]	PCM silicon based composite aggregate/Porous inclusion	Paraffin wax	PCM/Silicon (SiC) based composite aggregate re-placed with 30%, 50%, 70%, and 100% of the natural coarse aggregate.	Thermal conductivity	Gradually decreased with an increase of the replacement ratio of the PCM/SiC-based composite aggregate.
				Hydration heat development	Plain sample temperature was significantly increased compared with samples containing PCM/SiC-based composite aggregate during hydration process.

## 4. PCM Contribution to Achieve the UN SDGs

To align infrastructure development with the low-carbon-emissions target, indefatigable efforts have been put by the United Nations (UN) [114,115]. As a result, SDGs have been established as future goals for developed and developing countries. The UN SDG No. 7 is particularly devoted to affordable and clean energy, and PCMs have a significant role in promoting the use of clean energy [116,117]. The storage and utilization of clean energy with PCMs to achieve thermal comfort in building envelopes meet the different targets of UN SDG 7. Therefore, this section discusses the possible contribution of PCMs to the realization of different targets set under the UN SDGs. First, we emphasize the contribution of PCM to the attainment of the various target of UN SDG 7. Second, we map the use of PCM in concrete considering the UN SDGs. In addition, with the practical implementation of PCM in infrastructure practices, it is slightly challenging to accommodate this material with traditional practices. Therefore, a detailed discussion is presented of the required amendments of traditional construction practices to achieve the safe and quality use of PCMs at various stages of the construction process.

### 4.1. PCM Contribution to UN SDG 7

**Figure 9:**
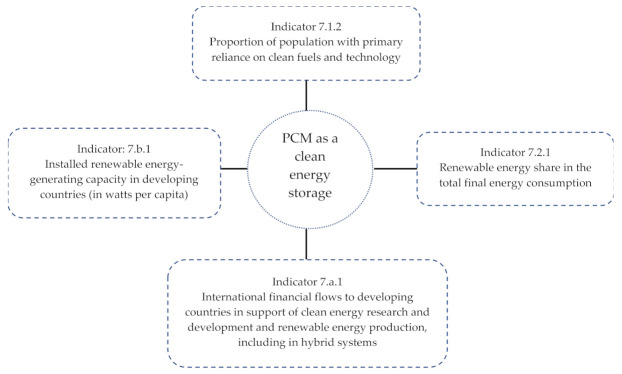
Contribution of PCM in attainment of UN SDGs 7.

UN SDG 7 refers to ‘affordable and clean energy’, with the agenda of providing access to clean energy research and technology, linked to renewable energy, energy efficiency, advanced and cleaner fossil-fuel technologies, investment promotions in energy infrastructure, expansions of infrastructure, and upgrades of technology to supply modern and sustainable energy services for all in developing countries, in particular in the least developed countries. The center of attention of UN SDG 7 is the achievement of clean energy for future development. The attainment of SDG 7 is measured by five targets. Each target is further subdivided into different sections, referred to as ‘indicators’. The literature reports that PCMs are among the efficient clean energy materials used in concrete and buildings to improve the thermal comfort of occupants [118]. The growing interest of researchers in PCMs is highly recognized for unfolding the unknown performance parameters of PCM concrete composites as thermal storage building materials [119]. Therefore, the implementation of PCM in a building material directly accelerates the efforts to attain the UN SDG 7. Figure 9 shows the relevance of PCM clean energy storage with the indicators of UN SDG 7.

Indicator 7.1.2 guidelines emphasize the use of efficient fuels and technology combinations in all major household energy end uses (e.g., cooking, space heating, lighting) to ensure health benefits. Indicator 7.2.1 shows the energy share out of the total final energy consumption levels. The energy share is specifically denoted as renewable energy in indicator 7.2.1. The renewable energy share out of the total final consumption is the percentage of the final consumption of energy that is derived from renewable resources. Further, the specific renewable energy sources defined in this indicator include the following sources of renewable energy: solar energy including solar PV and solar thermal energy; liquid biofuels including bio-gasoline, biodiesels and other liquid biofuels, solid biofuels including fuelwood, animal waste, vegetable waste, black liquor, bagasse and charcoal; and renewable waste energy covering energy from renewable municipal waste. Up to date, the study describing the direct measurement of UN SDGs attainment by using the PCM in the building materials has not been reported. Nonetheless, the indirect connection between the UN SDGs and PCM can be identified by literature related to energy-saving using PCM in the building component. Jangeldinov et al. [119] conducted a study on the implementation of PCM in a residential building constructed from lightweight steel. Eight different cities having a warm summer humid continental climate were selected. The maximum temperature reduction was 3.31 °C in the PCM implemented lightweight steel structure. The integration of PCM into the building framework saved the annual energy maximum up to 391.07 kWh. Olarte et al. [120] reported that the use of PCM in floor heating system save 18% energy cost. Zhang et al. [121] emphasize the use of PCM in the building for space heating and cooling of the building. The use of PCM for space heating and cooling of buildings helps in narrowing the gap between the peak and off-peak loads of electricity demand. The PCM based structural/non-structural elements use more solar energy and reduce the cooling load of air conditioning. Sharma et al. [122] reported that the PCM selection significantly depends upon the phase transition temperature, latent heat storage, and thermal conductivity. The high latent heat per volume unit PCM requires a lesser physical size of heat storage. Further, high thermal conductivity assists in the timely charging and discharging of energy storage upon demand. These findings present a benefit of using PCM in the building for promoting renewable energy consumption. The space heating and solar thermal applications described in indicators 7.1.2 and 7.2.1 reflect the strong connection between the PCM concrete composite/PCMs with the UN SDGs 7.

Additionally, indicator 7.a.1 about clean energy research and development depicts the section of solar energy research and emphasizes solar thermal applications and solar heating. Therefore, PCM also helps to attain indicator 7.a.1. Furthermore, the application of PCM in buildings for thermal comfort indirectly supports indicator 7.b.1. This indicator refers to the installed capacity of power plants that generate electricity from renewable energy sources divided by the total population of a country. The increase in use of PCM as a building material decreases fossil-fuel energy-based HVAC practices, indirectly supporting the reduction of the global energy demand. It can be observed that PCM and passive building envelopes could be a larger supplier of clean energy in the building sector for the achievement of the 2030 targets.

It should be noted here that the use of PCM in infrastructure development is also compatible with the target achievement of SDGs 9, 11, and 12 through its contribution. The possible impact of PCM usage on the traditional practices associated with buildings is comprehensively discussed in the next section.

### 4.2. Impact of PCM Usage on the Stages of Building Life Spans and Mapping with SDGs

**Figure 10:**
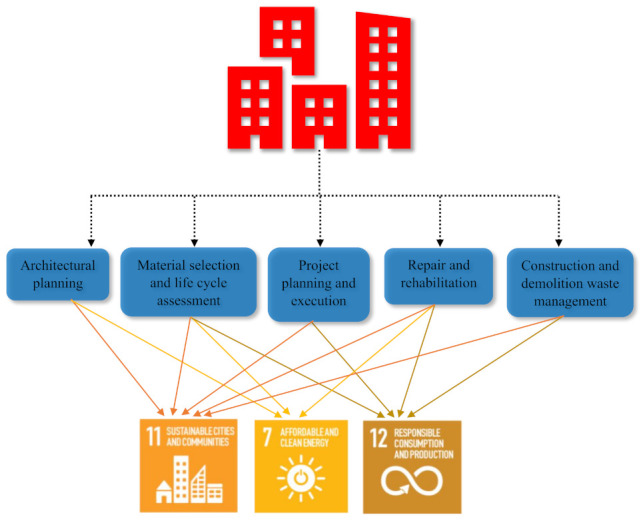
Mapping of various construction stages with UN sustainable development goals after considering the use of PCM in concrete.

A concrete composite must pass through various stages (architectural planning, material selection, planning and execution, repair and rehabilitation, construction and demolition) associated with the building lifetime. These stages are considered traditional infrastructure practices for the efficient use of concrete. However, the use of PCM into concrete as a thermal energy storage material affects these traditional practices, as tailored PCM-incorporated concrete may have different mixing and handling procedures. In addition, the durability of PCM-incorporated concrete is still in the early phase of research. Furthermore, the energy efficient repair and demolition strategies of PCM-incorporated concrete may receive much attention in the future. Therefore, the mapping of PCM-incorporated concrete considering the various stages of building lifetimes with the UN SDGs is a motivational source for researchers to pursue their research on the challenging issues related to this concrete that continue to hinder its use in field applications. Figure 10 shows the various construction stages that will be affected after the large-scale application of PCM-containing concrete in the field and the mapping with the different UN SDGs.

The architectural planning of a net-zero energy building meets UN SDGs 7 (affordable and clean energy) and 11 (sustainable communities and cities), as shown in Figure 10. The improvement in the energy efficiency of a building via the optimized design of building elements, shape, and geometry was also reported [123]. The energy-efficient walls such as Trombe walls, autoclaved aerated concrete (AAC) walls, double skin walls, and green walls were tested for their effect on the overall energy efficiency of the building [124]. For instance, the Trombe wall stores the solar thermal energy during the day and releases it inside the building overnight [125]. Odunfa et al. [126] investigated the effect of the orientation of a building on the energy demand. The north–south facing of the building was the most beneficial orientation for reducing the energy demand. The building oriented towards East–West shows the increase in energy demand. Raof [127] reported that the horizontal curve shape of buildings is equivalent to rectangular shape building and better than the vertical curve in cooling load. However, the architectural planning of PCM-incorporated infrastructure requires somewhat of a different approach than those used in the traditional practices due to certain limitations. The use of PCM in concrete can limit the freedom of concrete utilization under an adverse environment, special structural applications and complicate its placement in skeleton-like structure elements. The use of PCM increases the thermal inertia of the construction materials [128]. Therefore, the proportion of energy storage through PCM incorporation and sustainable infrastructure practices such as the orientation of the building, the geometry/shape, the window-to-wall area ratio, and shading devices for passive cooling and heating of the building envelope using solar energy [129,130,131,132,133,134,135] all need to be maintained.

Zang and Zang [136] conducted a study that compared the embodied emissions by different structural schemes considering the same building design of a residential building. Brick masonry, hollow block masonry, reinforced concrete frames, and reinforced concrete wall structures were analyzed. It was reported that the reinforced masonry and concrete frame structures were considerable options to control the embodied emissions. Additionally, hollow block masonry structures could be a good option for buildings in non-seismic regions considering their relatively low project costs and emissions. In terms of energy-saving building elements, double-skin facades (DSF) enable the separation of two surfaces with an air cavity. The DSF shows efficiency in maintaining high thermal comfort for occupants, but the efficiency is significantly altered depending on the type of window glazing, the design of the cavities, and the flows of air between them [137,138,139]. In addition, the use of effective electronic controllers, their installation at different locations can also help to maximize the use of renewable energy, consequently making life healthier for the user. However, it remains a concerning area of development for the building software development industry to create a virtual 3D model fully able to show a complete operational building. Simultaneously, the software calculates the energy costs along with local environmental factors. Therefore, it is challenging for the architects to promote the use of PCM-incorporated concrete on a large scale in the absence of authenticated data related to performance comparisons between this type of concrete and other available options in terms of strength, thermal energy storage, and durability performance. Research related to the long-term performance of PCM-incorporated concrete and the corresponding functions and interactions with the surrounding environment will support architects in their efforts to promote the use of PCM based on the structure type and local environmental conditions.

The material selection and life cycle assessment stages of infrastructure practices help to achieve UN SDGs 7, 11, and 12. However, the use of PCM as a building material in the infrastructure practices can lead to some interaction with the binding materials and can alter the fresh, mechanical and durability properties, as indicated in Table 2 [140]. PCM has been successfully applied in wallboards to store high levels of thermal energy in a building [23]. However, the incorporation of PCM into concrete remains a challenge, e.g., paraffin-based PCMs exhibit good stability and resist degradation in high pH environments, but are non-polar in nature; therefore, their bonding with concrete hydration products is not possible [141]. This observation significantly affects the material selection stage and its method of use in concrete. In addition, the scarcity of data on the durability and life cycle assessments of various types of PCM-containing concrete represents a massive challenge to those seeking to adopt this material in an actual project.

The execution of a building project with traditional practices is no longer sustainable due to governmental and non-government policies and given that society is concerned with sustainable practices of the construction industry [142]. The planning and execution stages are shifting towards optimum resource utilization, reduced waste generation, and lower CO_2_ emissions during the construction stages [143]. Therefore, the project planning and execution are mapped with UN SDGs 11 and 12. In the future, the use of PCM as a thermal energy storage material requires intense planning and execution skills, e.g., the handling and storage of PCM, skilled manpower at PCM concrete worksites, and quality assurances of PCM-concrete composites.

**Figure 11:**
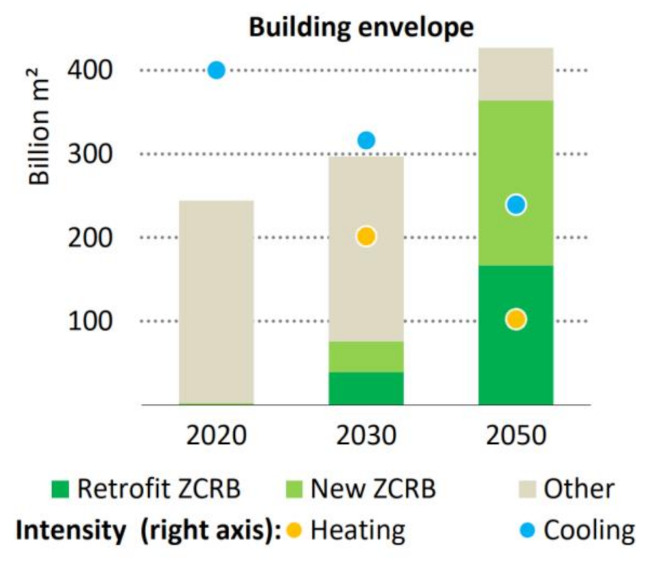
Targeted retrofitting of existing building to achieve zero carbon ready building and upcoming building following the zero-carbon-ready building energy codes (IEA, All right reserved) [128].

Achieving the decarbonization of energy use in the building sector requires almost all existing buildings to undergo a single in-depth retrofit by 2050, and new construction to meet stringent efficiency standards. Building energy codes covering new and existing buildings are a fundamental policy instrument to drive such changes. These energy codes are slated to be implemented in all regions by 2030 [144]. Thus, this implies an upcoming shift in the retrofitting of buildings, not only the strength upgrades but also towards net-zero carbon-ready buildings (ZCRB). ZCRB means that the buildings meet zero-carbon-ready building’s energy codes. Figure 11 shows that over 85% of the buildings are the ZCRB type with reduced levels of demand for heating and cooling by 2050. Hence, the application of PCM to upcoming building retrofitting projects for energy-efficient retrofitting cannot be ignored. PCM can be implemented in the built environment to raise the existing buildings category and transform them into a greener category of infrastructure. The integrated use of sustainable concrete and PCM to reduce CO_2_ emissions and the maximum utilization of thermal energy for heating and cooling purposes in buildings can be the future scope to attain the UN SDGs 7 (affordable and clean energy), 11 (sustainable communities and cities) and 12 (responsible consumption and production). However, comprehensive research is required to analyze the contributions of PCMs to energy-efficient retrofitting efforts to transform an existing structure into ZCRD structures.

The last stage associated with the building life is demolition and waste management. Table 3 shows the top nine countries’ demolition waste production with their population size and economic activities of the country. Furthermore, the evidence indicates that the construction industry generates approximately 44% of landfill waste in the United Kingdom, 44% in Australia, 40% in Brazil, 29% in the United States, 27% in Canada, and 25% in Hong Kong [145].

However, demolished waste consists of traditional components of concrete, steel, wood, and electric waste thus far. However, as PCMs used in infrastructure, soon demolition materials will contain PCM-concrete composites. This waste may affect the existing waste concrete reuse practice, such as the recycling of concrete paste and aggregate materials. The interaction between PCMs and concrete degrades the hydration products of concrete paste and may reveal new challenges when attempting to recycle this concrete. Thus far, no study has been conducted to assess the properties of concrete prepared using PCM-containing waste concrete material as a filler material.

Based on the above discussion, it can be concluded that every segment of the building process correlates with the environmental protection practices as recommended by the UN SDGs. Consequently, the weight/attention to think, plan, procure material, execute the project and supervise to attain durability, repair and retrofitting not only to achieve strength, but also for higher sustainability, to reduce the carbon footprint, and for the effective management of the construction and demolition waste management stages has increased. The reason for the increased weighting in each of the construction activities is due to the introduction of energy-efficient materials. Additionally, the outcomes of infrastructure and urban development for each country are now going to be measured in terms of the reduction of the carbon footprint and embodied energy. However, the regular practice of handling thermally efficient concrete for building construction will provide experience to practitioners associated with the different stages of building lifetimes, thus reducing the time required to make a zero-carbon-ready building fully functional.

## 5. Techniques for the Safe Deposit of PCM in Concrete for Large-Scale Applications

Presently, PCM-incorporated concrete is in the research and development stage. The work thus far on PCM has revealed the strength degradation of PCM-integrated mortar/concrete compared to that of normal mortar/concrete, as shown in Table 2. Overcoming the strength degradation issue of building materials using PCM is still a challenge. Another issue is long-term cyclic stability, which needs extensive research to establish PCM incorporation into the concrete/mortar as a durable option [147]. Due to the scarcity of sufficient research data, no technical guidelines related to PCM usage in concrete have been documented.

However, certain recent attempts, such as the coating of PCM containing LWA, the use of nanomaterials in PCMs, pipes filled with PCM, the use of supplementary cementitious materials in concrete to overcome the degradation effect of PCM, and PCM impregnation into concrete pores from the surface, all show performance improvements of PCM-containing concrete [91,148,149]. Jafarabad et al. [28] reported that the compressive strength was improved by 25% by using the silica fume in a cement matrix and polyethylene glycol as a PCM compared to a specimen without silica fume. Le et al. [150] revealed that the coating on PCM containing LWA with cement is an efficient method to improve the performance of concrete. Cement is chemically compatible with the bond development between the PCM host material and the paste phase. Therefore, the use of SCM or cement is better than the other types of coating materials. Royon et al. [151] reported an efficient method of increasing the thermal mass of buildings using a safe deposit of PCM in eight cylindrical holes of a concrete slab. Similarly, PCM deposited into PVC pipes and then embedded into the concrete pavement is an efficient technique to improve the performance of concrete [152]. Haider et al. [91] investigated the interaction of low-temperature phase change material with concrete prepared with lightweight aggregate. The normal aggregate was replaced with 50% and 100% epoxy coated and PCM impregnated lightweight aggregate. The compressive strength corresponding to 50% and 100% replacement was decreased by 28.94% and 38.82%, respectively, as compared to the control specimen. Furthermore, silica fume and carbon nanotubes have been added to the concrete to improve the properties of the paste phase. It was found that the addition of silica fume and carbon nanotube regain the strength by 13.30% in the case of 50% PCM-LWA mix and 18.82% in 100% PCM-LWA mix.

All of the aforementioned techniques are improvements over the direct inclusion of PCM in concrete and consequently imparts more reliability with regard to product performance outcomes in terms of the mechanical and durability properties. These properties are the foremost requirements for novel concrete composite products for large-scale applications. The large-scale application of PCM concrete composites will better serve the UN SDGs by using renewable energy. In addition to these assessments of the field experimental reliability of PCMs, the development of building modeling software that can handle the maximum parameters (e.g., humidity factor, outdoor temperature in different climates, the contribution of solar renewable systems with various types of solar energy, integrated modeling using PCM passive systems with natural night ventilation) to provide a unified solution for predicting the maximum benefits of using green materials and renewable energy still requires more effort regarding its hands-on use. Furthermore, direct evaluations of the output of building modeling scores with UN SDG infrastructure-related targets can be helpful to align the practitioner towards the measurement of the conservation of energy.

## 6. Current Challenges and Future Perspectives

Considering the positive impact of the use of PCM in building materials, the researchers are putting efforts on the more safe, durable, and reduction on the negative effect of PCM on the concrete mechanical properties. The following challenges limit the widespread use of PCM in concrete on field projects.

### 6.1. Thermal Performance of PCM

The low thermal conductivity of PCM decreases the rate of heat-absorbing/releasing during the phase change operation. Specifically, low thermal conductivity PCM is ineffective in energy storage when the temperature changes rapidly [147]. Additionally, the full potential of PCM is not utilized due to the increase of charging and discharging time of PCM [153]. The use of carbon foam, metal foam, layered clay minerals such as kaolin, carbon fibers, carbon nanotube, and nanoparticles to enhance the thermal conductivity of PCM have been reported in the past. The copper nanoparticles were dispersed into paraffin wax to improve the thermal conductivity. The size of the copper nanoparticle was 20nm. The maximum increase in synthesized copper-PCM was 46.3% when 2.0 wt.% of copper nanoparticles were dispersed in the PCM. The copper nanoparticles were also acted as a nucleation agent and reduced the supercooling effect during the phase change process [154]. The carbon-based additives were also added to the PCM to improve the thermal conductivity. The thermal conductivity of PCM increased up to 12 times when 5 wt.% graphite was added to the PCM [155]. However, the improvement in thermal conductivity of PCM is still in the research and development stage, and most of the PCM used in the infrastructure-related research is primarily from the family of paraffin wax and fatty acids. Therefore, the lack of data in the literature is available regarding the interaction of synthesized PCM with the cement matrix.

### 6.2. Trade-Off between Concrete Performance and PCM

Various literature reported that the concrete strength starts decreasing with the increment of PCM dosage in concrete [105,156,157,158,159,160]. The extent of strength degradation depends upon the method by which the PCM is incorporated in the concrete. Therefore, the direct incorporation of PCM in concrete is an obsolete method. To avoid the direct interaction between the PCM and concrete matrix, the porous aggregate filled with PCM and coated with epoxy is the feasible method of reducing the negative effect of PCM on the strength of concrete [161]. However, the building components primarily functioning as heat storage devices and strength is the secondary criteria such as wallboard, ceiling tiles, and floor panels, the use of PCM is successfully implemented [162]. These building components act as a non-structural component and the minimum performance requirement described by the ASCE 7 [163] is mandatory to achieve the operational performance of the building after an earthquake [164]. The failure of non-structural components disturbed the overall functioning of the building. The cost and time involved in the rehabilitation of the building are other important factors. Therefore, the seismic certification of PCM contained non-structural components is the current challenge.

### 6.3. Link between the Outcome of PCM Based Building Materials and UN SDGs

The SDG Report 2021 [165] reported that the annual energy efficiency improvement rate was 2% from 2010 to 2018. The required energy efficiency improvement rate is 3% to attain the targets for the 2018 to 2030 period. The report also emphasized the significant efforts are required on the safe and wide acceptability of modern renewable energy, especially in the heating and transport sectors. The share of renewable energy is increased by 4.27% in 2018 compared to the data of 2010. Presently, the electricity used in a building for heating and cooling space is primarily dependent on fossil fuels. However, the PCM uses renewable energy in buildings for heating and cooling purpose and decreasing fossil fuel- based electricity consumption. The methodology to reveal a link between PCM-based building materials outcomes and UN SDGs is not established so far. The direct measurement of the reduction in consumption of fossil fuel-based electricity after using the PCM products in a building from the field projects will motivate the policymaker, engineer, and society. The measurement of the outcome of PCM-based building materials helps architects and design engineers to directly measure and visualize the effectiveness of their selected building design and material selection contribution in the attainment of UN SDGs.

## 7. Conclusions

The present study reviews the PCM and its application in two aspects. The first aspect is the PCM type, properties, its application in the concrete, and the associated challenges of using PCM in concrete. The second aspect is the contribution of PCM-concrete composite to the UN SDGs.

Supplementary cementitious material and epoxy coated lightweight aggregate exhibit a safe deposit of PCM in concrete. The use of nanomaterials and silica fume in concrete recover the strength reduction of PCM concrete composites. The improvement in the paste phase using nanomaterials and silica fume is the cause of strength recovery. For the successful field application of PCM concrete composites, the trade-off between mechanical and thermal storage property remains a challenge for researchers. Quality assurance of composite matrixes by the complete elimination of PCM leakages into the matrix, good long-term cyclic stability, and a negligible negative effect on the compressive strength remain open and highly complicated areas of research.

PCM used in concrete can significantly contribute to the attainment of UN SDG 7 globally. The efforts have been made to map the available parameters (targets and indicators) present in the UN SGDs with the benefits of using PCM in concrete for infrastructure development. The complete lifespan of buildings is divided into five different stages (planning, material procurement, execution, repair and retrofitting, and construction and demolition waste). The contribution of each stage when using PCMs to the infrastructure-related UN SDGs 7, 11, and 12 is extensively presented. Undoubtedly, more scientific measurements are required to deliver a firm connection between PCM concrete composites and the UN SDGs.

In a nutshell, it can be concluded that the application of PCM on a large scale during the different levels of the building construction process has significant potential to achieve net-zero-ready buildings and can thus strongly contribute to the realization of the UN SDGs. This study disseminates the awareness of the PCM in the UN SDGs attainment on a global scale to the research community.

## Figures and Tables

**Table 3:** C&DW generation worldwide [145,146].

ID	Country	C&DW Generation (Million Tonnes)	Area (km^2^)	Population 2018 (Million)	GDP 2018 (Billion USD)
1	Hong Kong	20	1050	7.4	363
2	Australia	20.4	7,692,020	25	1434
3	Netherlands	22	33,690	17.2	914
4	Italy	39	294,140	60.5	2084
5	United Kingdom	58	241,930	66.5	2855
6	France	65	547,557	67	2778
7	Germany	86	349,360	83	3948
8	United States	534	9,147,420	327	20,544
9	China	1130	9,388,210	1393	13,608

## Data Availability

Data is contained within the article.
